# Left Ventricular Dimensions and Diastolic Function Are Different in Throwers, Endurance Athletes, and Sprinters From the World Masters Athletics Championships

**DOI:** 10.3389/fphys.2021.643764

**Published:** 2021-03-15

**Authors:** Fabian Hoffmann, Stefan Moestl, Savannah V. Wooten, Sten Stray-Gundersen, Corey R. Tomczak, Jens Tank, Hirofumi Tanaka, Jörn Rittweger, Philip D. Chilibeck

**Affiliations:** ^1^German Aerospace Center (DLR), Institute of Aerospace Medicine, Cologne, Germany; ^2^Department of Cardiology, University Hospital, Cologne, Germany; ^3^Department of Kinesiology and Health Education, The University of Texas at Austin, Austin, TX, United States; ^4^College of Kinesiology, University of Saskatchewan, Saskatoon, SK, Canada; ^5^Department of Pediatrics and Adolescent Medicine, University Hospital, Cologne, Germany

**Keywords:** older, elderly, lifelong exercise, aging, heart, track and field

## Abstract

There is controversy whether a lifetime of heavy resistance training, providing pressure-overload, is harmful for left ventricular function. We compared left ventricular dimensions and function in elite Masters athletes involved in throwing events (requiring strength; *n* = 21, seven females, 60 ± 14 years) to those involved in endurance events (*n* = 65, 25 females, 59 ± 10 years) and sprinting (*n* = 68, 21 females, 57 ± 13 years) at the 2018 World Masters Athletic Championships. Left ventricular dimensions and function were assessed with B-mode ultrasound and Doppler. The ratio of left ventricular early diastolic peak filling velocity to peak velocity during atrial contraction (E/A) across the mitral valve and the ratio of E to velocity of the E-wave (E’) across the lateral and septal mitral annulus (E/E’) were used as indexes of left ventricular diastolic function. Intra-ventricular septal wall thickness was greater in throwers compared to sprinters (11.9 ± 2.2 vs. 10.3 ± 2.3 mm; *p* = 0.01). Left ventricular end diastolic diameter/body surface area was higher in endurance athletes and sprinters vs. throwers (25.2 ± 3.0, 24.3 ± 3.1, and 22.0 ± 3.1 mm/m^2^, respectively, *p* < 0.01). The E/A was higher in endurance athletes and sprinters vs. throwers (1.35 ± 0.40, 1.37 ± 0.43, and 1.05 ± 0.41, respectively; *p* < 0.01). The E/E’ was lower in endurance athletes and sprinters vs. throwers (6.9 ± 1.8, 6.6 ± 1.9, and 8.1 ± 1.9, respectively, *p* < 0.05). Compared to age-matched historical controls (*n* > 1,000; E/A = 1.06; E/E’ = 7.5), left ventricular diastolic function was not different in throwers, but superior in endurance athletes and sprinters (*p* < 0.01). Masters throwers have altered left ventricular dimensions and function vs. other athletes, but a lifetime of heavy resistance training does not appear to alter left ventricular function compared to age-matched controls.

## Introduction

Aerobic endurance training in middle-age or older adults is able to alter cardiac function and might contribute to enhanced left ventricular diastolic function. Changes in heart morphology include eccentric hypertrophy of the heart, where ventricular dimensions and volumes are increased due to volume overload (D’Andrea et al., 2007b; Schmidt et al., 2014). On the other hand, resistance training results in acute increases in blood pressure and afterload (MacDougall et al., 1992) which may cause changes in heart structure over time, specifically a concentric hypertrophy, characterized by thickening of the walls of the left ventricle (D’Andrea et al., 2007b). Whether this affects left ventricular function is an active area of controversy. Only two studies have evaluated “master” athletes involved with long-term resistance training. D’Andrea et al. (2007b) showed that master resistance trained individuals (mean age 48 years; training duration > 10 years) had greater septal and posterior wall thickness in the left ventricle, but no difference was observed in left ventricular diastolic function compared with age-matched controls. In contrast, Haykowsky et al. (2000) found no differences between master athletes (mean age 46 years; mean training duration 18 years) at a national powerlifting championship compared to age-matched untrained controls for measures of cardiac dimensions or function. A 12-month longitudinal resistance training study in older men (age 68 years) likewise found no changes in cardiac structure or function (Schmidt et al., 2014).

Throwers involved in track and field events may represent different somatotypes than master resistance-trained athletes (such as powerlifters) previously evaluated. Because throwers would probably be larger individuals where absolute strength, rather than relative strength (i.e., relative to body mass) is more important, they may exhibit somatotypes that are more similar to those from “Strongmen” competitions. Younger Strongmen competitors (aged 25–45 year; mean 10 years of training) have greater absolute (i.e., not adjusted for body size) cardiac dimensions (left ventricular diameter, left ventricular wall thickness) than controls, but accompanied by reduced diastolic function (as assessed by a reduced ratio of early to late diastolic filling velocity; i.e., E/A), indicating that these individuals may be at greater risk for cardiovascular dysfunction (Venckunas et al., 2011).

Elite masters-level athletes are ideal to study because they would have been practicing intense training routines for long time periods; therefore, they can provide important information on how effects of aging on cardiac structure and function can be altered with chronic training. Our study represents a novel assessment of high-level track and field athletes drawn from the World Masters Athletic Championships and therefore some of the most highly-trained athletes in the world for their age groups. Previous studies evaluating master-level strength-trained athletes evaluated much smaller groups of athletes who were younger (i.e., mean age 46–48 years) than athletes in the current study and not trained at a “world-class” level as the athletes in the current study (Haykowsky et al., 2000; D’Andrea et al., 2007b).

The purpose of our study was to assess older athletes involved in throwing events at the World Masters Athletics Championships, to evaluate individuals who are older and with greater years of resistance training than those evaluated in earlier studies (Haykowsky et al., 2000; D’Andrea et al., 2007b). We compared these athletes to athletes involved in endurance events and those involved in sprinting events. A secondary purpose was to compare endurance athletes to sprinters, since only small groups of master endurance and sprint athletes have been compared previously for cardiac dimensions, with generally larger dimensions found in endurance athletes (Child et al., 1984). Our main hypothesis was that master athletes involved in throwing events would demonstrate higher left ventricular mass and ventricular wall thickness than those involved in endurance or sprint events. Based on research involving younger “strongmen” competitors, we also hypothesized that master athletes involved in throwing events would exhibit lower left ventricular diastolic function compared with those involved in endurance or sprint events and age-matched historical controls. Based on the volume overload typically experienced by endurance athletes, we hypothesized they would experience greater left ventricular diameter compared to the other athletic groups. The practical application of this study is that it allows determination of whether chronic (i.e., lifetime) training for strength, which involves pressure overload on the heart, is harmful for cardiac function.

## Materials and Methods

This was a cross-sectional study evaluating cardiac structure and function in athletes who participated in the 23rd World Masters Athletic Championship in Málaga Spain from September 4th to 15th, 2018. This was a sub-study as part of the Masters Athletic Field Study 2018 (MAFS-18) which was registered with the German Registry for Clinical Trials^1^ with study identifier DRKS00015172. The study was approved by the Masters Athletics Organizing Committee, the Ethics Commission of the North Rhine Medical Association, and the University of Saskatchewan Biomedical Research Ethics Review Board. Participants were informed of the experimental risks and signed an informed consent document prior to the investigation.

Inclusion criterion for the study was that participants had to be registered for the World Masters Athletic Championship. All data collection occurred onsite at the championships. Exclusion criteria were injuries or illnesses that affected jump tests or contraction of the calf muscle, another main outcome parameter of the MAFS-18. The time of day tested varied as data collection occurred throughout the day (i.e., usually from 8:00 am until 5:00 pm). The study included 21 throwers (14 men, seven women, from events including shot put, hammer throw, weight throw, javelin, discus, and throw pentathlon), 65 endurance athletes, which included athletes from long- and middle-distance events (40 men, 25 women, from events including marathon, 10,000 m, 5,000 m, steeplechase, 8 km cross-country running, walking events, 1,500 m, and 800 m) and 68 sprinters (47 men, 21 women, from events including 100 m, 200 m, 400 m, and hurdles. Long jumpers and triple jumpers were also included in this group). The group of throwers included 15 top-10 finishers (in their respective age group), five podium placements, and two world champions. The endurance group included 26 top-10 finishers, nine podium placements, and five world champions. The sprint group included 24 top-10 finishers, eight podium placements, and three world champions.

Cardiac structure and function were assessed with transthoracic echocardiography and Doppler imaging (Vivid-IQ with M5SC-RS sector probe, GE Healthcare, Boston, MA, United States) (Hoffmann et al., 2020). Two-dimensional-guided echocardiography was employed, using a parasternal long axis view, apical to the mitral valve leaflets and performed according to the American Society of Echocardiography Guidelines (Mitchell et al., 2019). All ultrasound procedures were performed and analyzed by the same sonographer (FH). Variables assessed included intra-ventricular septum thickness, left ventricular end-diastolic diameter (absolute and relative, corrected for body surface area), left ventricular posterior wall thickness, left ventricular mass (absolute and relative, corrected for body surface area), and relative wall thickness, calculated as 2 × posterior wall thickness divided by left ventricular end-diastolic diameter (Ford, 1978). Diastolic function was assessed with a multi-parametrical approach including transmitral peak filling velocities [early (E) and atrial (A) wave]the E/A ratio, deceleration time of the E-wave, tissue velocities corresponding to the E-wave (E’) derived from velocities in the lateral and septal mitral annulus, the E/E’ ratio, left atrial volume indexed to body surface area, and peak velocity of a present *trans-*tricuspid regurgitation jet (Nagueh et al., 2009). Ejection fraction was used as an indicator of global systolic function.

Training characteristics were assessed by questionnaire that asked about hours per week devoted to training during the competitive and off-seasons, hours per week devoted to training to build up muscle bulk (hypertrophy) and the length of the competitive season in months per year. The athletes were also asked (yes or no) whether they regularly included explosive weight training (e.g., explosive squats or deadlifts) and plyometrics (for half an hour or more each week) as part of their training routine.

All statistical analyses were performed using Statistica 7.0 (Chicago IL, United States). Comparisons between athletic groups (throwers vs. endurance athletes vs. sprinters) and sexes (males vs. females) were made using a 2-factor (3 × 2) between-groups ANOVA. Least squared difference *post hoc* analysis was used if there was a significant effect for athletic group or athletic group × sex interaction. The E/A ratio and E/E’ ratio, as indicators of global diastolic function were compared between each athletic group and means from age-matched historical controls (*n* > 1,000) (Watanabe et al., 2005; Caballero et al., 2015; Popović et al., 2018) using an independent samples *t*-test.

We also assessed differences across athletic groups and age by grouping athletes as ≥60 years (i.e., “older”) and <60 years (i.e., “younger”) and using an athletic group × age ANOVA. This age cut-off was chosen because it divided the throwing and endurance groups evenly. Sex was not included as a factor in this sub-analysis because the number of younger females in the group of throwers was considered too small (*n* = 2).

Within each athletic group we also compared cardiac structure and function variables between the top-ranked finishers (i.e., anyone who finished top six in their age group) and the rest of the athletes using a rank (top six vs. others) × sex (males vs. females) between-groups ANOVA. The top-ranked sprinters were significantly older than the other sprinters (64.0 ± 12.7 vs. 53 ± 14.7 years; *p* < 0.01); therefore, we used age as a covariate in this analysis.

For training variables, the percentage of athletes who indicated they regularly trained using explosive weight training or plyometrics was compared between athletic groups using Chi-square analysis.

All results are presented as means ± SD, except for frequencies of athletes who indicated (yes, no) whether they regularly used explosive weight training or plyometrics as part of their training (this was expressed as a percentage). Significance was accepted at *p* ≤ 0.05 for all statistical tests.

## Results

Participant characteristics are presented in Table 1. Throwers were heavier than sprinters and endurance athletes (*p* < 0.001) and sprinters were heavier than endurance athletes (*p* < 0.01). Throwers were taller than endurance athletes (*p* < 0.01). Throwers had greater body surface area compared to sprinters and endurance athletes (*p* < 0.001) and sprinters had greater body surface area compared to endurance athletes (*p* < 0.001).

**TABLE 1:** Participant characteristics.

	Throwers		Endurance athletes		Sprinters		Sex *p*-value	Athletic group *p*-value
	Males (*n* = 14)	Females (*n* = 7)	Males (*n* = 40)	Females (*n* = 25)	Males (*n* = 47)	Females (*n* = 21)		
Age (years)	58.6 ± 14.2	63.0 ± 13.6	60.8 ± 10.5	57.4 ± 10.2	57.5 ± 12.6	54.7 ± 13.8	0.83	0.23
Weight (kg)	95.2 ± 15.0	76.0 ± 13.4	68.1 ± 7.7	62.0 ± 21.6	74.7 ± 67.9	68.4 ± 23.9	<0.001	<0.001**
Height (cm)	182 ± 6	166 ± 3	172 ± 6	156 ± 22	175 ± 7	161 ± 25	<0.001	0.023*
Body surface area (m^2^)	2.19 ± 0.18	1.87 ± 0.18	1.80 ± 0.12	1.60 ± 0.11	1.90 ± 0.13	1.71 ± 0.12	<0.001	<0.001**

All values are means ± SD. There were no significant sex × athletic group interactions. *Throwers > endurance (LSD post hoc; p < 0.01). **Throwers > sprinters > endurance (LSD post hoc; p < 0.001).

Training variables for the different athletic groups are presented in Table 2. Athletic groups did not differ for total training volume (h/week) during the competitive or off-seasons. Volume of training devoted to muscle hypertrophy was significantly greater in the throwers and sprinters compared to endurance athletes (*p* < 0.01). A significantly greater proportion of throwers and sprinters indicated they regularly performed explosive weight training and plyometrics than endurance athletes (*p* < 0.001). The endurance athletes had a longer competitive season compared to sprinters (*p* < 0.05).

**TABLE 2:** Training characteristics.

	Throwers		Endurance athletes		Sprinters		Sex *p*-value	Athletic group *p*-value
	Males (*n* = 14)	Females (*n* = 7)	Males (*n* = 40)	Females (*n* = 25)	Males (*n* = 47)	Females (*n* = 21)		
Training volume during competitive season (h/week)	6.1 ± 3.4	8.0 ± 3.1	9.0 ± 5.0	9.4 ± 8.0	7.8 ± 3.7	9.9 ± 5.1	0.14	0.27
Training volume during off-season (h/week)	5.1 ± 3.3	7.6 ± 4.9	7.0 ± 4.4	8.8 ± 7.4	7.7 ± 5.2	8.1 ± 3.9	0.12	0.49
Training for muscle hypertrophy (h/week)	2.4 ± 1.1	3.3 ± 1.4	1.4 ± 1.7	1.8 ± 1.9	2.7 ± 0.8	2.2 ± 0.8	0.46	0.004*
Regularly do explosive weight training (% of participants)	43	57	10	24	57	43	0.95	<0.001*
Regularly do plyometrics (% of participants)	50	57	20	28	47	52	0.90	<0.001*
Competitive season (months)	6.1 ± 3.2	7.6 ± 2.3	7.2 ± 3.3	7.9 ± 3.9	6.3 ± 3.2	5.6 ± 0.1	0.47	0.033**

Values are means ± SD or%. There were no significant sex × athletic group interactions. *Throwers, sprinters > endurance (LSD post hoc; p < 0.01). **Endurance (sprinters (LSD post hocpost-hoc; p (<0.05).

**FIGURE 1:**
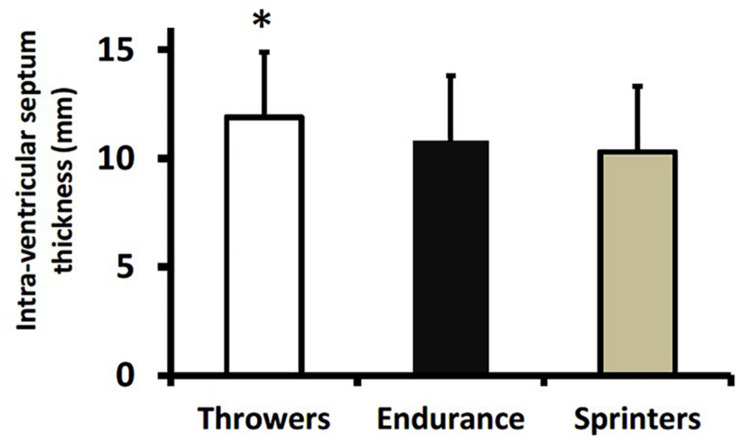
Intra-ventricular septum thickness in throwers, endurance athletes, and sprinters. Values are means ± SD. *p = 0.01 vs. sprinters.

**FIGURE 2:**
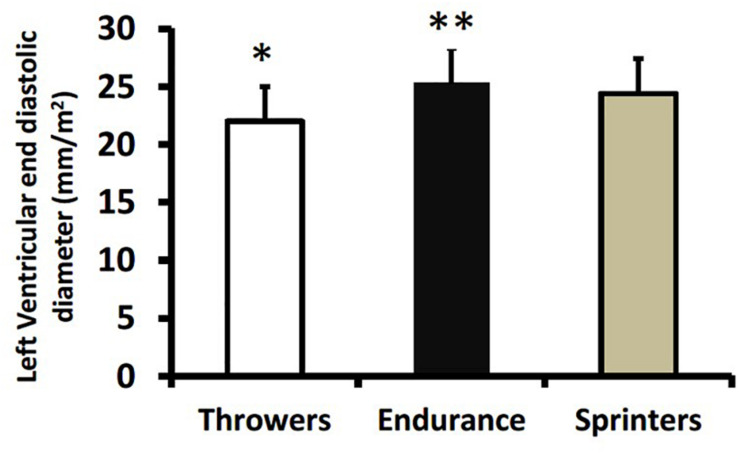
Left ventricular end-diastolic volume relative to body surface area in throwers, endurance athletes, and sprinters. Values are means ± SD. *p < 0.01 vs. endurance athletes and sprinters; **p < 0.05 vs. sprinters.

Table 3 presents values for left ventricular structure and function across athletic groups and sexes. There were no athletic group × sex interactions. Throwers had greater intra-ventricular septal thickness than sprinters (*p* = 0.01; Figure 1) and tended to have greater intra-ventricular septal thickness compared to endurance athletes (*p* = 0.054). Endurance athletes (*p* < 0.001) and sprinters (*p* < 0.01) had greater left ventricular end diastolic diameter when expressed relative to body surface area compared to throwers, and endurance athletes had greater relative diameter compared to sprinters (*p* < 0.05) (Figure 2).

**TABLE 3:** Cardiac structure and functional variables of elite athletes.

	Throwers		Endurance athletes		Sprinters		Sex *p*-value	Athletic group *p*-value
	Males (*n* = 14)	Females (*n* = 7)	Males (*n* = 40)	Females (*n* = 25)	Males (*n* = 47)	Females (*n* = 21)		
Intra-ventricular septum thickness (mm)	12.0 ± 1.7	11.7 ± 2.0	11.3 ± 2.7	10.3 ± 2.0	10.9 ± 1.8	9.7 ± 1.9	0.044	0.023*
LV diameter ED (mm)	46 ± 5	42 ± 3	44 ± 4	41 ± 5	45 ± 5	43 ± 5	0.003	0.36
LV diameter index (mm/m^2^)	21 ± 3	23 ± 3	25 ± 3	26 ± 3	23 ± 3	25 ± 3	0.009	<0.001^¶^
LV posterior wall ED (mm)	11 ± 2	10 ± 1	11 ± 2	11 ± 2	10 ± 2	10 ± 2	0.13	0.071
LV mass (g)	196 ± 45	159 ± 21	176 ± 47	144 ± 41	166 ± 39	138 ± 36	<0.001	0.070
LV mass index (g/m^2^)	89 ± 19	86 ± 12	98 ± 27	90 ± 27	87 ± 21	81 ± 21	0.22	0.081
Relative wall thickness (mm)	0.48 ± 0.10	0.50 ± 0.08	0.51 ± 0.12	0.52 ± 0.11	0.47 ± 0.10	0.47 ± 0.09	0.81	0.056
Left atrial volume index (mL/m^2^)	28 ± 9	21 ± 5	30 ± 11	28 ± 11	27 ± 10	28 ± 11	0.25	0.27
MV early filling (E) (cm/s)	66 ± 13	70 ± 18	63 ± 14	74 ± 19	69 ± 13	77 ± 23	0.021	0.27
MV atrial filling (A) (cm/s)	64 ± 14	69 ± 13	51 ± 17	57 ± 14	55 ± 18	57 ± 13	0.19	0.017**
E/A	1.07 ± 0.31	1.03 ± 0.26	1.34 ± 0.43	1.36 ± 0.38	1.35 ± 0.37	1.39 ± 0.43	0.87	0.01^¥^
Deceleration time of the E wave (ms)	238 ± 53	238 ± 40	216 ± 65	195 ± 50	191 ± 68	203 ± 92	0.84	0.09
E’ (cm/s)	9.5 ± 2.2	8.9 ± 3.9	10.1 ± 2.4	10.5 ± 2.6	11.0 ± 2.2	11.7 ± 2.4	0.73	0.004^¶^^¶^
E/E’	7.2 ± 1.4	9.0 ± 4.2	6.5 ± 1.6	7.3 ± 1.9	6.5 ± 1.6	6.7 ± 1.6	0.011	0.012**
Peak velocity of tricuspid regurgitation jet (m/s)	2.4 ± 0.4	2.3 ± 0.4	2.3 ± 0.4	2.5 ± 0.3	2.4 ± 0.3	2.2 ± 0.5	0.94	0.62
Ejection fraction-biplane (%)	60 ± 6	63 ± 3	61 ± 5	64 ± 5	60 ± 5	61 ± 6	0.043	0.24

ED, end-diastolic; LV, left ventricular; MV, mitral valve; E/A, ratio of early to late filling velocity across the mitral valve; E’, early diastolic annual velocity across the mitral valve. All values are means ± SD. There were no significant sex × athletic group interactions. *Throwers > sprinters (LSD post hoc; p = 0.01); Throwers > endurance (LSD post hoc; p = 0.054). **Throwers > endurance, sprinters (LSD post hoc; p < 0.05). ^¶^Throwers < endurance, sprinters (LSD post hoc; p < 0.01); Sprinters < endurance (LSD post hoc; p < 0.05). ^¶^^¶^Sprinters > throwers (LSD post hoc; p < 0.01); Sprinters > endurance (LSD post hoc; p < 0.05). ^¥^Throwers < sprinters, endurance (LSD post hoc; p < 0.01).

For all three athletic groups, we did not observe overt left ventricular diastolic dysfunction. Left atrial volume indexed to body surface area and maximal velocity of transtricuspid regurgitation, if present, neither differed between groups nor was elevated beyond a clinically relevant threshold (Table 3).

**FIGURE 3:**
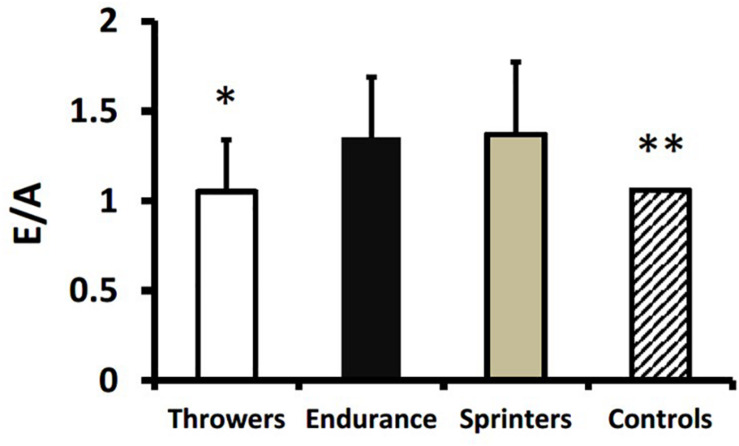
Ratio of early to late filling velocity (E/A) across the mitral valve in throwers, endurance athletes, sprinters, and historical controls. Values are means ± SD. *p < 0.01 vs. endurance athletes and sprinters. **p < 0.001 vs. endurance athletes and sprinters.

**FIGURE 4:**
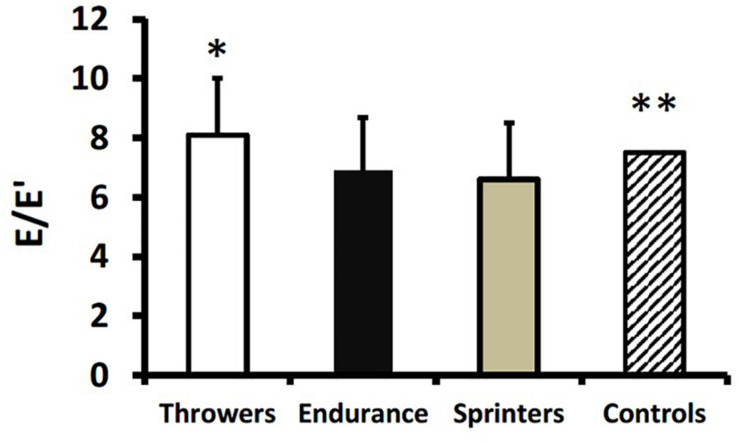
Ratio of early filling velocity across the mitral valve relative to velocity corresponding to the E-wave (E’) across the lateral and septal mitral annulus (E/E’) in throwers, endurance athletes, sprinters, and historical controls. Values are means ± SD. *p < 0.05 vs. endurance athletes and sprinters. **p < 0.01 vs. endurance athletes and sprinters.

Endurance athletes and sprinters had higher E/A compared to throwers (*p* < 0.01; Figure 3). The E/A for throwers was not different from historical controls; whereas endurance athletes and sprinters had higher E/A compared to historical controls (*p* < 0.001) (Figure 3). Although deceleration time did not differ between groups there was a trend toward longer times in throwers compared to endurance and sprint athletes (*p* = 0.09; Table 3). Averaged E’ from the lateral and septal mitral annulus was significantly higher in sprinters compared to throwers (*p* < 0.01) and endurance athletes (*p* < 0.05). The E/E’ ratio as a surrogate for left ventricular end diastolic pressure was lower in endurance athletes and sprinters compared to throwers (*p* < 0.05; Figure 4). Compared to age-matched historical controls throwers had similar E/E’ ratio; whereas E/E’ ratio was lower in endurance athletes and sprinters (*p* < 0.01; Figure 4).

Two athletes were excluded from the analysis because of relevant, yet asymptomatic aortic stenosis (*n* = 1) and relevant, yet asymptomatic aortic insufficiency (*n* = 1). Four subjects had to be excluded because of atrial fibrillation. We did not observe any mitral vitium that might have prohibited diastolic assessment. Athletic groups did not differ for ejection fraction, as a measure of systolic function (Table 3).

When athletic groups were divided into older (≥60 years) and younger (<60 years) groups, there were 11 older and 10 younger participants in the throwing group, 32 participants each in the older and younger endurance groups, and 28 older and 40 younger participants in the sprint group. There was no significant athletic group × age interactions, indicating that changes with age were similar across athletic groups. The older group had greater septum thickness (11.9 ± 2.3 vs. 10.4 ± 2.4 mm; *p* < 0.01), left ventricular posterior wall thickness (11.0 ± 2.0 vs. 10.3 ± 2.1 mm; *p* < 0.05), left ventricular mass (180 ± 46 vs. 157 ± 50 g; *p* < 0.01), left ventricular mass indexed to body surface area (97 ± 24 vs. 83 ± 26 g/m^2^; *p* < 0.01), and E/E’ ratio (7.6 ± 0.9 vs. 6.5 ± 2.2; *p* < 0.01), and lower E/A ratio (1.09 ± 0.41 vs. 1.39 ± 0.42; *p* < 0.01).

When the top six finishers from each athletic group were compared to the rest of the athletes, there was no difference within the throwers for any measure of cardiac structure and function (data not shown). For endurance athletes, the top-ranked athletes (*n* = 16) compared to the other athletes (*n* = 49) had greater left ventricular end-diastolic diameter (absolute: 45.8 ± 4.4 vs. 42.0 ± 4.4 mm; indexed to body surface area: 27.7 ± 2.7 vs. 24.2 ± 2.6 mm/m^2^; both *p* < 0.01), and left ventricular mass indexed to body surface area (108 ± 26 vs. 91 ± 26 g/m^2^; *p* < 0.05). There was a rank × sex interaction for E/E’ ratio, where the top-ranked endurance females (*n* = 7) compared to the rest of the endurance females (*n* = 18) had a lower ratio (6.2 ± 1.7 vs. 7.8 ± 1.7; *p* < 0.05). For sprinters, the top-ranked athletes (*n* = 14) compared to the rest of the athletes (*n* = 54) had lower E/E’ ratio (adjusted for age; 5.8 ± 1.5 vs. 6.8 ± 1.6; *p* < 0.05), but no differences in cardiac structure.

## Discussion

The most important finding from our study was that masters athletes involved in long-term training for strength and endurance demonstrate unique cardiac adaptations that might be expected from their training characteristics. For example, resistance training is characterized by a pressure overload *via* high blood pressures experienced acutely during exercise (MacDougall et al., 1992). This is proposed to result in a concentric hypertrophy where the walls of the heart become thicker without cardiac dilation. There was some evidence for wall thickening where intra-ventricular septum thickness was greater in throwers than in sprinters (Table 3 and Figure 1). On the other hand, aerobic endurance training is proposed to involve a volume-overload and eccentric hypertrophy (Schmidt et al., 2014). Again, there was some evidence for this from our study where endurance athletes had greater left ventricular diameter (adjusted for body surface area) compared with throwers and sprinters (Table 3 and Figure 2). It should be noted that our results for endurance athletes are specific to track and field sports involving mainly running and walking. Past research with endurance athletes involved in different postures (i.e., rowing or canoeing) indicate that they might experience different adaptations to cardiac structure; specifically a thickening of the ventricular wall (Pelliccia et al., 1991). A greater volume-overload in our endurance athletes and sprinters might result in a greater volume-flow over the mitral valve and can be seen as a surrogate for enhanced diastolic function, as the E/A ratio was greater in endurance athletes and sprinters compared to throwers and age-matched historical controls (Figure 3). Furthermore, throwers had the lowest value for E’ which is an important surrogate for ventricular compliance or restriction. We hypothesize, that with highest values for E/A-ratio in endurance trained athletes their hearts are trained to provide high cardiac output over a longer time period without the need of maximum contractility; whereas sprinters with the highest values in E’ use peak contractility for maximum diastolic function over a shorter duration. Throwers’ performances are not limited by oxygen delivery due to the extremely short movement time (1–5 s) and its reliance on directly available intracellular ATP stores; therefore, they do not seem to profit from enhanced diastolic function compared to age-matched controls. It is important to note any morphological differences in heart structure of athletes involved in throwing events did not result in lower diastolic function compared to age-matched historical controls (Figures 3, 4), which is different from our original hypothesis based on younger strongmen competitors (Venckunas et al., 2011).

An unaltered left atrial volume index between athletic groups (Table 3) is surprising given that there were differences between groups for diastolic function. Especially in endurance trained athletes we expected elevated values of left atrial volume index as Abdulla and Nielsen (2009) stated that chronic endurance training is correlated with enlarged left atria and an increased risk of atrial fibrillation. Furthermore, murine animal models show that chronic daily exercise can lead to left atrial dilation, scarring and adverse remodeling (Guasch et al., 2013). However, in our cohort we only had to exclude three individuals with atrial fibrillation. With his prevalence of 1.9% in our cohort we are well below the estimation of the European Society of Cardiology of 2–4% in adults (Hindricks et al., 2020). Chronically altered transmitral filling patterns, as evidenced from the higher E/A in our endurance and sprint athletes can result in changes in left atrial morphology. However, this is dependent on preload status (Alarrayed et al., 2009) as well as left ventricular compliance (Greenstein and Mayo, 2018). Although we could not observe differences in left ventricular mass index between disciplines, endurance athletes had larger left ventricles (i.e., left ventricular-diameter index) whereas throwers had a thicker myocardium (i.e., intraventricular septal thickness). This difference might lead to a relatively reduced ventricular compliance in throwers compared to endurance athletes without crossing the threshold of clinical relevance and not yet altering left atrial morphology.

When comparing athletes across age groups, there were similar changes across age within each athletic group (i.e., there were no athletic group × age interactions). Higher age was associated with a thicker septum and left ventricular posterior wall and increased left ventricular mass, as well as lower cardiac function as indicated by higher E/E’ and lower E/A ratios, changes which are expected with age (Watanabe et al., 2005; Caballero et al., 2015; Popović et al., 2018). These changes with age, however, were not different between athletic groups.

When comparing athletes who finished in the top six in the world for their age group to the remaining athletes, there were no differences for cardiac structure and function within the group of throwers, indicating that their adaptation to training is quite consistent across elite vs. less elite athletes. In contrast, within the endurance and sprint groups the more successful athletes (i.e., top six finishers within age groups) had differences in either structure or function compared to their less successful counterparts. Within the endurance group, top six finishers had greater left ventricular end diastolic diameter and left ventricular mass (relative to body surface area) compared to the rest of the athletes, indicating that volume overload was especially experienced by these athletes. These changes in cardiac structure are similar to previous studies of elite master endurance athletes (Bohm et al., 2016). The top female endurance athletes and the top sprint athletes had lower E/E’ ratio as a surrogate for left ventricular end diastolic pressure, indicating that the most elite athletes in these groups had superior diastolic function.

Because throwers train for maximum absolute strength and power (i.e., with no limit on body size), we had hypothesized that they might have similar heart structure and function as younger “strongmen” competitors. Younger “strongmen” competitors (age 35 years) have similar intra-ventricular septum thickness (∼12 mm) compared to the Masters throwers in our study (Table 3). Younger strongmen also had greater intra-ventricular septum thickness and lower relative left ventricular end-diastolic diameter compared to marathon runners (Venckunas et al., 2011) again similar to our results for throwers compared to endurance athletes (Table 3 and Figure 2); however, unlike our Masters throwers, younger strongmen had reduced diastolic function as assessed by E/A compared to age-matched controls (Venckunas et al., 2011). The younger strongmen competitors in the study by Venckunas et al. (2011) had larger body surface area than the throwers in our study (i.e., 2.5 vs. 2.2 m^2^ for the males in our study); therefore, perhaps their training for absolute strength was of greater intensity. In addition, throwers need to train for power in addition to strength; therefore, training routines may differ. Throwing involves movement with maximal velocity; therefore, there may be some limits on body size for successful performance. Within the throwing group, hammer and javelin throwers are usually smaller than throwers in other events, but when we compared these groups, there were no differences in body mass or body surface area (data not shown). Another factor for differences in diastolic function could be that strongmen competitors are generally not limited in anabolic steroid use (Venckunas et al., 2011) whereas the athletes at the Masters World Athletic Championships were tested for doping. The World Masters Athletics Championships follows the International Association of Athletics Federations guidelines^2^, which state: “Athletes to be tested shall be selected as follows: (a) on a final position basis and/or random basis, and/or; (b) at the discretion of the Anti-Doping and Monitoring Committee by any method that it shall choose, including target testing, and/or; (c) any athlete who has broken or equaled a World Masters Athletic Championships World Record.” Chronic anabolic steroid use is associated with reduced diastolic function, as indicated by lower E/A ratio (D’Andrea et al., 2007a). Despite having altered cardiac structure compared to endurance-trained athletes and sprinters, the Masters throwers in our study had similar left ventricular diastolic function compared to age-matched historical controls.

Resistance-trained athletes of younger age than the throwers in our study (i.e., aged 46–48 years vs. ∼59 years) had measures of cardiac structure that were varied compared to the throwers in our study. Haykowsky et al. (2000) showed that Masters powerlifters had intra-ventricular septum measures not different from controls and lower (i.e., 9.4 mm) than the throwers in our study and resistance-trained athletes from D’Andrea et al. (2007b) (i.e., ∼12 mm). The sport of powerlifting includes weight categories; therefore, training might differ whereby most of these athletes would train for relative strength (i.e., to keep their body mass to a minimum) while throwers from our study would be training for maximum strength, without any limit on body size. D’Andrea et al. (2007b) compared their resistance-trained athletes (age 48 years) to endurance athletes (swimmers) with very similar results compared to our study. Their resistance-trained athletes had greater intra-ventricular septum thickness compared to endurance athletes; whereas our throwers had greater intra-ventricular septum thickness compared to sprinters (Figure 1). The resistance-trained athletes from D’Andrea et al. (2007b) had lower left ventricular end-diastolic diameter compared to their endurance-trained group; whereas our throwers had lower left ventricular end-diastolic volume (relative to body surface area) compared to our endurance-trained group (Figure 2). Finally, the A/E ratio, as an indicator for diastolic function, was very similar across studies with the resistance-trained Masters athletes from D’Andrea et al. (2007b) having identical values to our throwers (1.05) which was lower than endurance athletes (∼1.3 from both studies), but not lower than age-matched controls. Our throwers, despite being about 10 years older, have very comparable cardiac structure and function to “younger” resistance-trained athletes.

Our cross-sectional results for Masters throwers and endurance athletes are partially supported by one longitudinal training study in older males (aged 65–75 years). Twelve months of endurance training (i.e., soccer) increased left ventricular end-diastolic diameter and enhanced E/A ratio, with no changes with resistance training (Schmidt et al., 2014). Unlike our Masters throwers however, 12-months of resistance training did not increase intra-ventricular septum thickness. Perhaps longer durations of resistance training with higher pressure overload is needed for these changes. It should be noted that sprinters and endurance athletes from our study still had markedly higher E/A ratio (∼1.36) compared to the older males from the study of Schmidt et al. (2014) after 12 months of training (E/A ratio = 1.11) providing support that years of endurance or sprint training may have a greater effect for improving diastolic function.

A unique aspect of our study was the inclusion of Masters sprint athletes. Only one previous study involving small groups compared master sprinters (*n* = 13; mean age 47 years) to endurance (*n* = 9; mean age 54 years) athletes (Child et al., 1984). This study showed that master track endurance athletes had larger cardiac dimensions relative to body surface area (including left ventricular mass, posterior wall and septal thickness, and left ventricular end diastolic diameter) than master sprinters (Child et al., 1984). Surprisingly, in our study the sprinters had similar cardiac adaptations compared to endurance athletes, with larger relative left ventricular end-diastolic diameter and superior diastolic function (i.e., higher E/A ratio and lower E/E’ ratio) compared to throwers and age-matched historical controls (Figures 2–4). Sprinters usually include some resistance training as part of their program; however, they would need to train more for relative strength because carrying a larger body mass would be a disadvantage for their sport. There is a very obvious difference in somatotype (i.e., significantly lower body surface area for sprinters, Table 1) between sprinters and throwers. We speculate that training for a very lean body composition for sprinters may offer a training stimulus more similar to that experienced by endurance athletes (i.e., volume overload) than throwers (i.e., pressure overload). It has been noted that master sprinters may incorporate a significant amount of endurance-type training into their routines and that sprint-interval type training may confer cardiovascular benefits (Kusy and Zieliñski, 2015). This is supported by the finding from cross-sectional studies that sprinters may actually have better maintenance of maximal aerobic capacity over their lifespan compared to endurance athletes (Kusy and Zieliñski, 2014). Sprinters’ training routines may therefore confer similar benefits regarding cardiovascular structure and diastolic function compared to the training routines of endurance athletes.

The strength of our study was the multi-parametric approach for comprehensive quantification of diastolic function since diastolic function is a clinical state which is complex to assess (Nagueh et al., 2009). Transmitral inflow patterns show strong dependency on cardiac preload and therefore volume status (Ie et al., 2003). Furthermore, different cardiac dysfunctions, such as intra-cardiac shunts, relevant mitral stenosis or insufficiency as well as overt heart failure may prohibit diastolic assessment (Nagueh et al., 2009). In our cohort, we did not observe clinical manifestation of hypervolemia or heart failure. We identified two relevant vitia which were excluded from the analysis; therefore, our data represents a valid quantification of diastolic function in the different groups.

Another strength of our study was inclusion of female Masters athletes, as there is a lack of exercise physiology research in female athletes compared to males (Costello et al., 2014). We are not aware of any other studies that have evaluated cardiac structure and function in female Masters athletes involved in sports requiring high strength, such as throwing. A recent study found that lifelong endurance training in females (>60 years of age) resulted in greater left ventricular mass index and superior cardiovascular function (i.e., greater static left ventricular chamber compliance) compared to age-matched untrained controls (Carrick-Ranson et al., 2020), similar to the results for female endurance athletes from our study. Overall, female athletes in our study had some differences compared to males (i.e., lower intra-ventricular septum thickness, lower absolute, but higher relative left ventricular end-diastolic diameter, and lower left ventricular mass), which were expected based on differences in body size. There was, however, no sex × athletic group interactions, indicating that females had similar differences across athletic groups as males.

A limitation of our study was that we were not able to use more advanced echocardiographic techniques such as 3-dimensional analysis or speckle-tracking analysis. This is a direction for future research. Although we assessed athletes across older and younger age groups within our study, we did not determine menopausal status in our female athletes. It would be of interest to track female athletes as they go through the menopause transition to determine the effect on cardiac structure and function.

In summary, our study indicated left ventricular structural differences between athletic groups, most likely due to their different modes of training (i.e., pressure overload for throwers vs. volume overload for endurance athletes and sprinters). More specifically, throwers had highest intra-ventricular septum thickness; whereas endurance athletes and sprinters had higher relative end-diastolic ventricular diameter. These may result in differences between athletic groups for diastolic function, which was superior in endurance athletes and sprinters, but not impaired in throwers compared to age-matched historical controls. Surprisingly, sprinters had similar left ventricular structure and diastolic function compared to endurance athletes, indicating adaptation to their training programs may be more similar to endurance training than resistance training. The practical application of our study is that although a lifetime of training for strength and power appears to affect cardiac structure (specifically a thickening of the intraventricular septum), it does not appear to negatively affect left ventricular function.

## Data Availability Statement

The raw data supporting the conclusions of this article will be made available by the authors, without undue reservation.

## Ethics Statement

The studies involving human participants were reviewed and approved by Ethics Commission of the North Rhine Medical Association, and the University of Saskatchewan Biomedical Research Ethics Review Board. The patients/participants provided their written informed consent to participate in this study.

## Author Contributions

PC, CT, and JR: study planning. FH, SM, PC, SW, SS-G, and JR: data collection. PC: manuscript writing. All authors edited the manuscript.

## Conflict of Interest

The authors declare that the research was conducted in the absence of any commercial or financial relationships that could be construed as a potential conflict of interest.

## Footnotes

1 www.drks.de

2 https://world-masters-athletics.com/documents/anti-doping-2/
